# Beyond modular and non-modular states: theoretical considerations, exemplifications, and practical implications

**DOI:** 10.3389/fpsyg.2025.1456587

**Published:** 2025-01-23

**Authors:** Francesco Benso, Carlo Chiorri, Eleonora Ardu, Paola Venuti, Angela Pasqualotto

**Affiliations:** ^1^Department of Psychology and Cognitive Science, University of Trento, Trento, Italy; ^2^Department of Education Sciences, University of Genoa, Genoa, Italy; ^3^Associazione Neuroscienze Cognitive Clinica Ricerca Intervento (ANCCRI), Genova, Italy; ^4^Faculty of Psychology and Education Sciences (FPSE), University of Geneva, Geneva, Switzerland; ^5^Department of Education and Learning, University of Applied Sciences and Arts of Southern Switzerland, Manno, Switzerland

**Keywords:** massive modularity, executive control, working memory, central executive network, neural networks

## Abstract

The concept of modularity in neuropsychology remains a topic of significant debate, especially when considering complex, non-innate, hyper-learned, and adaptable modular systems. This paper critically examines the evolution of cognitive modularity, addressing the challenges of integrating foundational theories with recent empirical and theoretical developments. We begin by analyzing the contributions of Sternberg and Fodor, whose foundational work established the concept of specialized, encapsulated modules within cognitive processes, particularly in the domains of perception and language. Building on this, we explore Carruthers’ theory of massive modularity, which extends the modular framework to broader cognitive functions, though we reject its application to central amodal systems, which are overarching and resistant to modularization. We also evaluate recent discoveries, such as mirror neurons and the neural reuse hypothesis, and their implications for traditional modularity models. Furthermore, we investigate the dynamic interactions between the Default Mode Network (DMN), Central Executive Network (CEN), and Salience Network (SN), highlighting their roles in shifting between automatic and controlled states. This exploration refines existing theoretical models, distinguishing innate systems, genetically predisposed ones, and those hyper-learned through working memory, as exemplified by the three-level model of Moscovitch and Umiltà. We address the blurred boundary between domain-specific and domain-general systems, proposing modular versus non-modular states—indexed by automaticity and mandatoriness—as key discriminators. This systematization, supported by empirical literature and our own research, provides a more stable framework for understanding modular systems, avoiding interpretive confusion across varying levels of complexity. These insights advance both theoretical understanding and practical applications in cognitive science.

## 1 Introduction

Modularity, the concept that cognitive processes are managed by subsystems that operate largely independently, has been central in cognitive science and neuropsychology for several decades (D’Souza and Karmiloff-Smith, 2011). Classical modularity theories, such as those proposed by Fodor (1983), emphasized that cognitive systems—particularly those involved in perception and language—are domain-specific, automatic, and encapsulated from other cognitive processes. However, this rigid view has been increasingly challenged by evidence from cognitive neuroscience and neuropsychology, suggesting a more dynamic and interconnected architecture of the mind (Barrett and Kurzban, 2006; Anderson, 2007, 2010).

Despite decades of debate, consensus on the definition of a “module” remains elusive. The term ‘modularity’ has been applied to various cognitive processes, from innate systems like acoustic frequency discrimination to complex, learned tasks like lexical access and metaphonological processing. This broad application has led to differing interpretations of the concept, as highlighted by Mahon and Cantlon (2011).

Modularity has also been extensively studied in various cognitive functions, such as perception. For instance, symmetry perception is a fundamental aspect of visual processing, playing a crucial role in object recognition, mate selection, and predator avoidance. Empirical evidence suggests that symmetry detection may be supported by specialized neural mechanisms, which could be considered modular in nature (Giannouli, 2013). Research indicates that certain visual areas in the human brain, such as the lateral occipital cortex, are particularly responsive to symmetrical patterns, suggesting a degree of functional specialization within the perceptual system (Sasaki et al., 2005). Studies on symmetry perception in animals, such as insects and birds, further support the idea that this perceptual ability may be underpinned by modular systems (Swaddle and Cuthill, 1994). These findings align with the broader concept of modularity in cognitive functions, where specific neural circuits are dedicated to processing particular types of information, allowing for efficient and rapid responses to environmental stimuli (mandatariety). While symmetry perception may initially operate in a modular fashion, higher-level cognitive processes, such as aesthetic judgment or decision-making, likely involve interactions with other cognitive systems, thereby reducing the strict modularity of the perceptual system. This example illustrates how modular processes can interact with more flexible, integrative systems, challenging the notion of strictly encapsulated cognitive functions.

The model we propose partly originated in the literature from Moscovitch and Umiltà (1990), and we have often discussed and used it to predict reading acquisition and treatment (see paragraph 6). Over time, we have further integrated it by conducting our own experiments (e.g., Benso and Umiltà, 1998; Pasqualotto et al., 2024), but we have also engaged extensively with studies on modularity across various disciplines (Barrett and Kurzban, 2006; Anderson, 2007, 2010). We have drawn from multiple authors considered experts in the field and focused on the February 2011 special issue on modularity published by Cognitive Neuropsychology.

Massive modularity studies followed (Carruthers, 2006; Ritchie and Carruthers, 2010), which we have cautiously accepted. Some aspects of these theories align with our own findings (Benso and Umiltà, 1998) in neural networks (i.e., some areas are common across circuits supporting different functions; see paragraph 3.2). However, we decisively distance ourselves from the idea of modularizing central systems (amodal and superordinate to various, even complex, systems). Additional theories, such as those of mirror neurons (Rizzolatti and Craighero, 2004) and neural reuse (Anderson, 2010), have also reinforced the shift away from the Fodorian paradigm.

Moreover, neuroanatomical studies of brain circuits have provided stable and hard-to-refute evidence (Op de Beeck et al., 2008; Friston and Price, 2011; D’Esposito and Postle, 2015; Menon, 2015), opening the door to a more complex modularity where functions are supported by entire brain circuits rather than by specific areas. The networks interact to form new abilities (such as reading and writing) required by human development. Individual areas act as hubs within larger networks that support multiple functions, diverging from Fodorian criteria such as *hardwiring, rigid encapsulation, non-assemblability, and mandatory processing.*

Therefore, the complex modular theory has been reorganized, starting from the Moscovitch and Umiltà (1990) model. We have defined different layers of modularity: (1) *innate*, (2) *genetically predisposed to development*, and (3) *hyper-learned through executive attention and working memory*. We later considered studies highlighting the Salience Network (SN), the Default Mode Network (DMN), and the Central Executive Network (CEN) (Dosenbach et al., 2007; Menon and Uddin, 2010; Sridharan et al., 2008). These innovations have strengthened our hypothesis, which stems from the study of observable behavior (especially in movement and sports), distinguishing modular states (governed by automatism and mandatory processing) from non-modular states (governed by the CEN and executive attention/working memory; see Table 1).

**Table 1:** Key concepts and definitions.

Concept	Explanation
Modularity	Cognitive processes that traditionally operate independently within specific domains.
Massive Modularity	The idea that even higher-level cognitive functions are modular but remain flexible and interconnected.
Neural Reuse	Brain systems can be reused for new functions, adapting to different tasks.
Gradient of Modularity	Modularity is not all-or-nothing; processes can be more or less modular depending on expertise and context.
Automaticity	A process is considered automatic when it meets at least two conditions: insensitivity to load and non-intentionality.
Mandatory Operations	A mandatory process triggered by innate reflexes or highly learned material.
Hardwired	A system that is hardwired, directly and exclusively connected to specific brain areas.
Encapsulation	A system that operates independently with autonomous computations, unaffected by external influences.
Evolutionary Psychology	A theoretical approach that examines cognition and behavior from a modern evolutionary perspective.
CEN	Central Executive Network, responsible for processes like executive attention and working memory.
SN	Salience Network, which triggers adaptive behavior and controls the switch between CEN and DMN.
DMN	Default Mode Network, active during low-attention states or negative emotional states, disrupting performance.
Hub	Key nodal centers in brain circuits indicating areas crucial for specific functions
Double Dissociation	Double dissociation occurs when one individual or group (e.g., Patient A) performs significantly better on Task I compared to another individual or group (e.g., Patient B), while the reverse pattern is observed for Task II, indicating that the two tasks rely on distinct and independent cognitive or neural mechanisms.
Abductive Inference	Abduction is a reasoning process that begins with observed outcomes and works backward to find possible causes. Unlike deduction, which draws conclusions from premises, abduction generates hypotheses to explain an effect, often used in science, medicine, and problem-solving. This reasoning is probabilistic, relying on testing hypotheses to confirm or reject possible explanations. For example, while “if Sylvester is a feline, then he is an animal” is true, “if Sylvester is an animal, then he is a feline” is a fallacy. Abduction is central to scientific inquiry, allowing researchers to explore multiple causes, generate theories, and advance understanding through hypothesis testing, even when certainty is not guaranteed.

We also reviewed more recent literature, where scholars have highlighted challenges to modularizing central systems (Lundie, 2019; Zerilli, 2019). We support this critique, considering two key explanations: first, frequent reductionist attempts to isolate and modularize executive functions (Rabbitt, 1997; Bernstein and Waber, 2007; McCabe et al., 2010; Benso, 2018; Benso and Chiorri, 2023). Second, evidence from various studies highlights the amodal and superordinate characteristics of central systems, which are clearly distinguishable from even complex modules (Engle and Kane, 2004; Cohen and D’Esposito, 2016; Yeon et al., 2024).

The failure to distinguish between high-level central processes and modularity can generate confusion, as several articles reference both massive modularity and Evolutionary Psychology theories (see Table 1; Sulikowski, 2016; Pietraszewski and Wertz, 2022; Egeland, 2024). Additionally, many authors call for greater differentiation in gradients of complexity between modules (Egeland, 2024; Pietraszewski and Wertz, 2022), as the lack of clarity in defining ‘module’ can lead to confusion when referring to diverse systems.

We employed abductive reasoning to develop the theoretical framework of this paper, selecting the most plausible explanations based on evidence from our experiments and interdisciplinary research (Coltheart, 2011). This method resulted in testable hypotheses and conclusions that draw from both literature and our own findings. The approach presented here—a differentiated systematization of studies and experimental results—is grounded in this reasoning framework. In scientific inquiry, abductive reasoning plays a fundamental role, as it allows for the selection of the most plausible explanation based on available evidence (Copi and Cohen, 1990; Peirce, 1997). It represents the inverse reasoning process typical of experimental science, where one starts with the results and initial weak inferences, formulates hypotheses for verification, and traces the causes back to their origin. While initial conjectures may be subject to fallacies, the iterative process of testing and refinement transforms them into scientifically valid hypotheses. Our systematization integrates insights from multiple fields, including art, sports, and clinical practice, recognizing the complexity of cognitive functions at play. Fodor (2001), in his discussions on modular theory, underscores the importance of grounding arguments in abductive inferences, a sentiment echoed by Barrett and Kurzban (2006). As the saying goes, “Where there is doubt, there is science; where there is certainty, science has ceased to operate.”

To provide a comprehensive overview of the theoretical foundations and evolution of modularity theories, we have summarized key frameworks, their defining features, strengths, limitations, and relevance to the current work in Table 2.

**Table 2:** Summary of modularity theories and their evolution.

Modularity theory	Key features	Strengths	Limitations	Our perspective
Fodor (1983)	Rigid encapsulation, domain specificity, mandatory processing. No assemblability hardwired	Provides a clear descriptive framework for basic systems.	Does not account for complex or integrated systems. While the criteria of modularity have their own logic, they are not applicable to the systems identified by Fodor (1983), such as input systems and language.	Useful for simple, innate modules (Level 1, e.g., reflexes and detection of frequencies) but not applicable to higher-order systems.
Moscovitch and Umiltà (1990)	Introduced three hierarchical levels of modularity: (1) innate, (2) genetically predisposed, and (3) hyper learning (assembled modules with executive attention).	It provides an organized taxonomy of modular systems, distinguishing them based on the unique characteristics of the three levels and the gradient of complexity.	It does not address dynamic modularity, However, the model was developed in 1990, whereas the most significant works on state changes (modular vs. non-modular) emerged after 2000.	Provides a strong foundation for stratified modular models, enriched with recent neuroscientific insights.
Massive Modularity (Ritchie and Carruthers, 2010)	Suggests modularity extends to complex, high-level central systems.	Highlights overlapping yet independent circuits (double dissociations). These independent circuits explain the phenomenon of ‘double dissociations,’ as shown in the neural network studies by Benso and Umiltà (1998).	Overgeneralizes modularity to central systems without clear evidence. We have distanced ourselves from this latter theorization, aligning with other scholars such as Barrett and Kurzban (2006), Lundie (2019), Zerilli (2019), and Cohen and D’Esposito (2016).	Supports some aspects (e.g., neural reuse), but we limit its application to avoid overextension.
Neural Reuse (Anderson, 2007, 2010)	Brain circuits are reused for multiple functions rather than being task-specific.	Explains multifunctionality of regions like the Visual Word Form Area.	Risks blurring the concept of separable modules and of “double dissociations.” From our perspective, the robustness of research on ‘separable modifiability’ (Sternberg, 2011) and ‘double dissociations’ (Shallice, 1988) remains undeniable, as even Ritchie and Carruthers (2010) have argued.	Supports neural reuse for certain processes but emphasizes preserving the distinction of modules.
Gradient Modularity (Barrett and Kurzban, 2006)	Cognitive processes exist on a spectrum from modular to non-modular, depending on task demands and the level of expertise.	It accounts for flexibility and varying degrees of modularity, allowing for a gradient of mandatoriness (automaticity; Yantis and Jonides, 1990; Cohen et al., 1990; Turatto et al., 2000; Benso, 2018).	May lack clear boundaries for module definitions.	Aligned with our perspective of a continuum between modular and non-modular states.

## 2 Modular theories from Fodor to Carruthers

Modularity has been a central topic in cognitive science, with significant contributions from various theorists. Among the most influential are Jerry Fodor and Saul Sternberg, whose perspectives have shaped our understanding of how cognitive processes are organized (Coltheart, 2011). These foundational ideas set the stage for Peter Carruthers’ theory of massive modularity.

Fodor (1983) proposed that certain cognitive functions are carried out by specialized, domain-specific modules. These modules are characterized by several key features: they are non-assemblable, informationally encapsulated, operate automatically, are fast, and are innate and hardwired, meaning they are linked to specific brain areas. In other words, a module is a closed system, characterized by informational impenetrability, meaning it processes information independently of other cognitive functions. Fodor’s model primarily focused on perceptual and linguistic processes, which he argued were managed by these hardwired, domain-specific systems, each designed to compute material specific to its designated domain. According to Fodor, these modules operate independently of other cognitive processes and are not influenced by an individual’s beliefs or knowledge.

Sternberg provided a different approach to understanding modularity, particularly focusing on the evaluation of functional independence between cognitive processes. Sternberg’s (2001, 2011) theory introduced the concepts of “separable modifiability” and “additive factors.” He argued that if two cognitive processes can be shown to be influenced by different factors, and these influences do not interact, then the processes are functionally independent and can be considered modular. This method allows researchers to determine whether certain cognitive processes operate independently of one another, thus providing a more empirical basis for identifying modules within the mind.

Carruthers extended the concept of modularity to include higher cognitive functions, proposing the idea of “massive modularity.” He argued that the mind is composed of a large number of specialized modules, not just for basic perceptual processes but also for more complex cognitive tasks. Carruthers’ theory suggests that these modules, while domain-specific, can interact and share information, thus offering a more flexible and dynamic view of cognitive architecture. This perspective challenges Fodor’s stricter view of modularity, suggesting that even central cognitive functions could be modularized, albeit in a less rigid, more distributed manner.

In their comprehensive review of modular theories, Mahon and Cantlon (2011) highlighted the lack of convergence on key issues within modularity research. They pointed out that the term “modularity” has been applied to a wide range of cognitive processes, leading to a diversity of interpretations and, at times, confusion in the literature. This diversity underscores the need for a more refined and systematic approach to understanding modularity, particularly in complex cognitive systems where traditional definitions may fall short (Barrett and Kurzban, 2006).

### 2.1 Modularity evaluated with neuroimaging

In functional neuroimaging, the identification of activations in distinct brain regions does not automatically confirm the separate modifiability of the cognitive processes underlying those activations. As Op de Beeck et al. (2008) pointed out, it is challenging to define clear boundaries for activations within specific brain areas. Additionally, findings from lesion studies have raised significant doubts about the notion of “pure subtraction”—the assumption that following a lesion, all cognitive functions would continue to operate as before, except for those reliant on the damaged area. Over time, neuroscience research has increasingly questioned the strict one-to-one correspondence between complex cognitive functions and particular brain regions.

In the context of modular theories, Mahon and Cantlon (2011) observed that assigning highly specific functions to neural processes using functional neuroimaging can be problematic. Friston and Price (2011) discussed how the dissociation of brain regions through neuroimaging may demonstrate separate modifiability, but it does not necessarily prove whether those regions are either necessary or sufficient for the underlying cognitive processes. Lesion studies further complicate the picture, as the concept of “dynamic diaschisis,” where a lesion in one part of the brain disrupts functions in other, seemingly unrelated areas, suggests that cognitive processes may be more interconnected and less modular than previously thought (Friston and Price, 2011). The same authors also emphasized that functional imaging alone cannot definitively establish the necessity or sufficiency of a brain region for a specific cognitive process, further complicating the interpretation of neuroimaging data in the context of modularity. Lurie et al. (2024) provide evidence of preferential functional connectivity between cortical areas, even non-adjacent ones, that are linked through the emission of similar and coordinated frequencies. In other words, as D’Esposito and Postle (2015) suggest, entire circuits must be considered, rather than individual areas with specific functions. Despite these challenges, neuroimaging has contributed to our understanding of modularity by revealing that certain brain regions do exhibit some degree of functional specialization. For example, the Visual Word Form Area (VWFA) in the left lateral occipitotemporal sulcus is consistently activated during reading tasks, supporting the idea of a specialized module for word recognition (Dehaene and Cohen, 2011). However, this specialization does not imply that the VWFA operates independently of other cognitive processes; rather, it suggests a more nuanced view of modularity where specialized functions are integrated within broader cognitive networks. Several studies have identified the VWFA as one of the possible “hubs” within larger circuits, suggesting that it is functionally connected to the dorsal attention network (Vogel et al., 2012). A more in-depth analysis presents a complex model of VWFA function characterized by two distinct circuits for integrating language and attention, pointing to connectivity-constrained cognition as a key principle of human brain organization (see Chen et al., 2019, who found these structural and functional aspects through Diffusion MRI and fMRI data, respectively). This evidence supports the idea that reading is not a closed system solely related to linguistic evolution; instead, it comprises multiple components—linguistic, visuospatial, and attentional—that have become modularized through hyperlearning, supported by executive attention (including working memory), as outlined by Moscovitch and Umiltà (1990) in their model (see below) and as recently shown in a study by Pasqualotto et al. (2024).

### 2.2 The need to revisit Fodorian modular characteristics in reference to complex functional systems

Fodor’s modularity theory, with its focus on domain-specific, encapsulated modules, provided a powerful framework for understanding basic cognitive processes. However, as cognitive science has progressed, it has become clear that this framework is insufficient for explaining the complexity of higher cognitive functions.

As cognitive systems become more complex, the characteristics that define Fodorian modules—such as encapsulation and automaticity—tend to degrade. For example, the notion of “informational encapsulation” becomes less tenable when considering complex tasks that require the integration of information across different cognitive domains. Studies have shown that even perceptual processes, which Fodor considered the epitome of modularity, can be influenced by higher cognitive functions like attention and expectation, demonstrating top-down penetrability (Stokes and Bergeron, 2015; Benso, 2018).

Barrett and Kurzban (2006) argue that absolute modularity in complex systems would be maladaptive, as it would preclude the flexibility needed for effective cognitive functioning. They propose a gradient model of modularity, where cognitive processes exhibit varying degrees of modularity depending on the task and the level of expertise. This perspective aligns with Sternberg’s concept of separable modifiability, which acknowledges that while some cognitive processes may operate independently under certain conditions, they often interact and share resources in more complex tasks. This perspective suggests that cognitive modules are not static but can evolve and adapt, becoming more or less modular in response to environmental demands.

Given these considerations, it is essential to revisit and revise the Fodorian concept of modularity when discussing complex cognitive systems. Instead of viewing modularity as a binary characteristic, we should consider it as a continuum, where different cognitive functions exhibit varying degrees of modularity depending on factors such as complexity, context, and learning.

In summary, while Fodor’s theory of modularity provided a foundational framework for cognitive science, it is increasingly evident that a more nuanced understanding is necessary to account for the complexity of cognitive systems. Revisiting and revising these concepts in light of new empirical evidence and theoretical developments is crucial for advancing our understanding of the mind’s organization. Within the realm of complex modularity, three of Fodor’s (1983) proposed characteristics remain relevant: domain specificity, encapsulation, and mandatory operations (automaticity). However, these terms take on entirely different interpretations in the context of complex modular theories. The concept of “domain specificity” is better understood as “functional specialization” (Dehaene and Cohen, 2011). For example, we assert that reading, when viewed as a complex module, comprises multiple specialized domains (for a complex module, the term ‘domain specificity’ is no longer applicable, as it requires the integration of multiple specialized domains). Similarly, encapsulation and mandatory operations (automaticity) should not be interpreted according to Fodor’s binary framework (all or nothing), but rather as existing on a continuum that varies depending on the level of expertise achieved through learning (Cohen et al., 1990; Petrov et al., 2010).

## 3 Beyond Fodor’s modular theory

We believe that additional lines of research should be considered for their contributions to expanding theories on complex modularity, including: (1) the discovery of mirror neurons, (2) the theories of ‘neural reuse,’ and (3) the theory of massive modularity. These studies will primarily be assessed to demonstrate the limitations of Fodor’s theory in accounting for complex modularity. For further clarification of the key concepts and definitions discussed in this section, see Table 1.

### 3.1 The discovery of mirror neurons

Neurophysiological and neuroimaging studies, such as those examining the mirror neuron system (MNS) (Rizzolatti and Sinigaglia, 2006), provide compelling evidence for the development of modular theories that extend beyond Fodor’s criteria. These studies support the notion of interactive integration between various cognitive processes. For example, Skipper et al. (2007), using fMRI, demonstrated an association between speech and gestures in Broca’s area. The MNS, which is a network of multimodal brain areas, has been found to be activated in a wide range of behaviors, including reflexive responses and the comprehension and production of complex actions (Rizzolatti et al., 2014). The MNS has also been suggested to be a pivotal component of a larger associative network (Traxler, 2013). In addition, the MNS has been found to underpin complex actions (Molnar-Szakacs and Overy, 2006), language (Rizzolatti and Craighero, 2004), and emotional expressions (Bastiaansen et al., 2009).

This evidence challenges the traditional view of a closed, independent, and disembodied modular linguistic system that manipulates amodal symbolic representations. To accommodate these findings, some authors have proposed different interpretative solutions, such as partially abandoning modular models for more complex functions (e.g., Anderson, 2010; Lundie, 2019). Gallese (2007) noted that studies on the MNS and language provide a different perspective from the widely accepted idea that language is a modular system functioning independently, manipulating symbolic representations without interaction with other cognitive processes.

### 3.2 The “neural reuse” hypothesis integrated into the concept of “massive modularity”

The hypothesis of “massive modularity” emerges from evolutionary psychology theorists, who suggest that evolutionary pressures shaped our minds to consist primarily of specialized mechanisms that operate over domain-specific representations (Cosmides and Tooby, 1994). However, this idea has faced significant criticism, especially regarding the proposed modularization of central systems. Scholars have argued that central systems, which oversee executive functions, cannot be modularized in the same rigid way as peripheral systems. This critique aligns with more recent findings in cognitive neuroscience, which suggest that many cognitive processes rely on overlapping brain areas (Barense and Lee, 2021; Kvasova et al., 2024).

The theory of “neural reuse” offers a more flexible model that integrates with and refines massive modularity. Neural reuse proposes that the brain reuses existing neural circuits for multiple functions, rather than evolving entirely new structures for each task (Anderson, 2010). This theory complements the idea of massive modularity, especially in non-innate systems, by demonstrating how the same brain regions can be redeployed across different cognitive functions. For example, Anderson (2014) suggested that the brain reuses behavioral, neural, and environmental resources for new cognitive capacities, a view supported by empirical evidence on the brain’s adaptive flexibility (Anderson, 2010; Raja and Anderson, 2019). The “neural reuse” hypothesis complements massive modularity by suggesting that brain areas originally evolved for specific functions are reused for new tasks (Anderson, 2010). According to Anderson (2014), the brain relies on pre-existing neural resources rather than developing new structures, thereby supporting multiple cognitive capacities through shared circuits. This theory suggests that areas in the brain can perform more than one function, complicating the strict notion of functional modularity.

Carruthers (2006) acknowledged that even systems designed for specific domains often need input from other systems. This integration of information across domains highlights the flexible, interconnected nature of cognitive functions. Anderson’s “massive redeployment” hypothesis (2007) extends this by arguing that the same neural circuits can support multiple functions. However, this view has faced criticism for its broad generalizations, with some authors (e.g., Brincker, 2010; Zerilli, 2019) questioning its explanatory power.

Despite these critiques, Ritchie and Carruthers (2010) argued that massive modularity aligns with neural reuse, explaining phenomena like “separable modifiability” (Sternberg, 2011) and “double dissociations” (Shallice, 1988). They posit that two cognitive functions can share overlapping brain areas but still operate independently. If non-overlapping parts are disrupted, only one function is affected, supporting the idea of partially modular systems.

Benso and Umiltà (1998) demonstrated this through neural network experiments, showing that double dissociation can occur in hyper-learned functions. Lesioning certain neurons led to impairments in both functions, while other neurons, when lesioned, selectively impaired one function without affecting the other. This provided clear evidence of double dissociations, where distinct neural circuits could support separate cognitive functions while still sharing some common processing resources.

These findings challenge Fodor’s rigid modular model, which assumes encapsulated, independent systems. Instead, they suggest a more dynamic form of modularity, where shared circuits support multiple cognitive tasks. This work partially foreshadowed the concept of massive modularity, as later articulated by Ritchie and Carruthers (2010), and partially anticipated the definition of massive modularity illustrated above by Ritchie and Carruthers (2010) years later. Recent evidence suggests that brain regions representing specific content, such as low-level visual features in early visual regions, are involved in processing these features across a wide range of cognitive processes, not just perception (Barense and Lee, 2021; Kvasova et al., 2024). This further supports the neural reuse hypothesis, where the same neural circuits are recruited for various tasks, irrespective of the specific cognitive demands. For example, visual features processed in early visual regions are consistently activated during both perceptual and memory tasks, demonstrating how cognitive processes rely on overlapping neural substrates rather than dedicated, isolated modules (Cowell et al., 2019; Pacheco-Estefan et al., 2024).

### 3.3 Further observations on the theories of “neural reuse” and massive modularity

Anderson’s (2010) hypothesis that true double dissociations do not arise in neuropsychology or are not valid separable modifiability experiments, while having supporting elements, needs much more evidence than Carruthers’s (2006) model (“principle of parsimony”). Although there are overlaps in functional areas, neuroimaging evidence, such as from the Visual Word Form Area (VWFA), provides compelling support for functional modularity.

For example, Dehaene and Cohen (2007, 2011), following their “neuronal recycling” hypothesis, demonstrated that reading systematically activates the left lateral occipitotemporal sulcus (VWFA), which is typically dedicated to recognizing faces and objects. Their findings revealed a strong correlation between VWFA activation and reading proficiency, with poor readers showing diminished activation in this area compared to object recognition tasks. In contrast, proficient readers exhibited robust VWFA activation during reading. This highlights the potential for using neuroimaging diagnostics to identify reading impairments, as reduced VWFA activity could indicate dyslexia or other reading difficulties. Consequently, these findings offer valuable insights for designing interventions that focus on activating or compensating for VWFA deficiencies, linking theory directly to clinical practice.

From an anthropological and biological perspective, these observations support the hypothesis that no gene for reading-writing exists, as such skills would have only emerged relatively recently—between 3,000 and 6,000 years ago—in *Homo sapiens*, whose history spans approximately 200,000 years. However, the VWFA illustrates how neural reuse facilitates the modularization of this recently developed skill.

These studies indicate gradual modularization processes in complex systems like reading, where the criterion of innateness does not apply. Furthermore, the observation that multiple circuits converge on a single area to support diverse functions aligns with the concept of complex modularity, diverging from Fodorian principles of *hardwiring* and *non-assemblability*. Neuroimaging thus offers a crucial bridge between theory and clinical applications by revealing how cognitive functions can be supported by adaptive neural systems and guiding targeted interventions for conditions like dyslexia.

Having provided sufficient evidence to justify our proposed dynamic modularity model, we now turn to addressing the theoretical rigidity often associated with *encapsulation* and *mandatory operation* (automaticity).

### 3.4 Complex modules: a gradient of encapsulation (impenetrability) and Mandatoriness (forced automatic response)

As Petrov et al. (2010, p. 287) aptly state, “Modularist terminology forces a binary distinction on what is fundamentally a continuum.” In the context of complex modular theory, it’s essential to recognize that top-down penetrability, reciprocal interaction, and graded automaticity are all integral aspects. These elements vary depending on the task and the individual’s level of expertise, as Lundie (2019) highlights the flexibility required by central systems. In line with this view, Kvasova et al. (2024) provided evidence for the gradient of modularity by demonstrating how different cognitive tasks engage varying degrees of modularity depending on the task complexity and the individual’s expertise.

Excluding central systems from modularization, complex modules—sensitive to context or internal emotional-motivational systems—cannot be entirely encapsulated or strictly mandatory. The concept of encapsulation has long been debated, particularly given solid evidence demonstrating top-down influences even on perceptual systems traditionally considered modular by Fodor (1983). For example, studies by Stokes and Bergeron (2015) and Benso (2018) confirm the top-down influence of attention on perception, with earlier work by Hillyard et al. (1973) and Woldorff et al. (1993) showing similar effects even during the earliest stages of visual processing. Moran and Desimone (1985) further demonstrated how attention modulates activity in V4 neurons, effectively filtering irrelevant stimuli. Interestingly, some studies indicate that top-down influences may enhance, rather than disrupt, modular efficiency (McAdams and Maunsel, 1999). Encapsulation is often associated with mandatory processing, where highly automated systems tend to become closed off. However, professionals, especially in fields like sports and the arts, recognize the need to deconstruct and adapt movements to maintain flexibility. Over-specialization and rigid patterns can lead to stereotyped responses, limiting creativity and adaptability. Yet, a certain degree of mandatory processing is a hallmark of expertise, contributing to the modularity of complex systems. Therefore, the concept of mandatory processing must be considered within the specific context of the system’s characteristics.

For a clearer outline of the modular taxonomy and neuropsychological evidence supporting each level, see Tables 3, 4.

**Table 3:** Reorganizing modularity levels.

Modular taxonomy: three levels—as described by Moscovitch and Umiltà (1990)	Examples and neuropsychological Evidence	Developmental characteristics	Modularity criteria
First-level modules	Visual and auditory frequency perception, motor reflexes.	Basic modules, genetically innate.	Full encapsulation, domain specificity, mandatory operation including all those proposed by Fodor (1983).
Second-level modules	Object recognition, language, walking. Impairment: Agnosia, aphasia.	Genetically predisposed for modularization but requires environmental inputs.	Partial encapsulation and mandatory operation; domain specificity,
Third-level modules	Reading, complex motor skills (e.g., dancing). Impairment: Dyslexia, alexia, apraxia, agraphia.	Experientially assembled; involves hyper-learned volitional processes. Resources in Working Memory are fundamental to development.	A gradient of Automaticity and encapsulation based on expertise, and task complexity, “functional specialization.”
There is no theorized aspect of modularization for high-level Central Systems (e.g., Executive Attention and Working Memory).			

**Table 4:** Modular and non-modular states (Benso, 2018).

State	SN-CEN-DMN intervention	Positive behavioral effects	Negative behavioral effects
Complex modular state	The CEN is deactivated once expertise is achieved, with routine behavior becoming reliant on expertise and operating as mandatory.	Expertise freely expressed (e.g., tennis swing, playing the piano, fluent handwriting).	Fear or excessive emotional responses can trigger CEN control, disrupting the fluidity of the expert performance.
Non-modular state	The CEN is engaged during learning, retraining, or control operations in response to emotional demands from the SN.	Slower processing under CEN control for re-learning or correcting automatic processes. This includes controlling actions and behavior in dangerous situations, as well as linguistic exercises involving spoonerisms.	Emotional deactivation of the CEN occurs when it should remain active (e.g., during tests or crossing roads), allowing emotional DMN intrusions.

### 3.5 Clarifications on mandatoriness

The literature on automaticity and mandatoriness in complex modules often presents these concepts dichotomously, which differs from our approach. Barrett and Kurzban (2006, 2012) argue that absolute mandatoriness in complex systems would be maladaptive, leading to inefficiencies and preventing the flexible use of computational resources. They also suggest that strict encapsulation, as envisioned by Fodorian modularity, would result in a computational explosion, where every relevant system produces output for every stimulus. To address this, a “gradient of modularity” is proposed, acknowledging varying degrees of automaticity in complex systems.

Even highly expert systems, such as those of tennis players, pianists, or readers, are never fully automated. This is evident in the need for constant practice to maintain proficiency, highlighting the positive aspect of variability in response to changing contexts. The gradient of automaticity that governs mandatoriness (forced automatic response), which correlates with the level of expertise, underscores the importance of flexibility within modular systems, distinguishing them from the rigidity typically associated with Fodorian modules. Research on attention, such as that by Turatto et al. (2000), supports this perspective. They refine Hasher and Zacks’ (1979) criteria for automatic processes, emphasizing that true automaticity—*insensitivity to load* and *non-intentionality*—is rare. This aligns with Yantis and Jonides’ (1990) findings on attention, suggesting that automatic processes are not as all-encompassing as traditionally thought. Moreover, the correlation between automaticity and modular complexity is evident in how even highly trained dancers or pianists experience degradation in skill without regular practice.

Cohen et al. (1990) observed that automaticity is not an “all-or-none” phenomenon, but rather that automatic processes are “continuous” and can also be subject to attentional control. Thus, a system can be considered modular if it reaches a degree of mandatoriness that minimizes the need for executive attention. However, this boundary is inherently fuzzy, influenced by the complexity of the task and the individual’s expertise but it remains an important reference point for navigating the confusion between modular and non-modular systems, as well as domain-specific versus general systems.

In conclusion, the concepts of encapsulation and mandatory processing have been reinterpreted to align with the idea of complex modularity. This perspective enriches our understanding of complex functional systems and allows us to classify different types of modules according to Moscovitch and Umiltà’s (1990) theory.

## 4 A modular hierarchical theory for organizing stratification

According to Mahon and Cantlon (2011), there is general consensus on the differentiation between low-computation innate systems and more complex, functionally distinct learned systems. The concept of a “module” encompasses systems that, although belonging to the same domain, function in fundamentally different ways. For example, reflexive blinking, walking, and dancing are all motor modules, yet they represent different levels of modularity. Reflexive blinking is genetically innate; walking is genetically predisposed to modularization (Karmiloff-Smith, 1994), developing implicitly over time, as suggested by Moscovitch and Umiltà (1990). In contrast, dancing involves hyper-learned, volitional processes that rely on executive attention and working memory, similar to tasks like reading.

Moscovitch and Umiltà (1990) introduced a modular typology with three hierarchical levels. First-level modules are genetically innate and include tasks such as acoustic and visual recognition or motor reflexes (this is the only level where the criteria of Fodorian modularity would be applicable). Second-level modules are genetically predisposed and arise by combining first-level modules with implicit attention, playing an essential role in functions like object recognition and language. Third-level modules involve volitional processes, emerging from the integration of second-level modules with explicit executive attention, as exemplified by activities such as reading and complex motor skills like dancing. This typology underscores a clear differentiation between innate systems, which align with Fodor’s (1983) description of strict modularity, and those systems that become modularized over time through experience and learning, as described by Karmiloff-Smith (1994). This modular theory, which considers a gradual increase in complexity, has also been applied to the motor domain (Rizzolatti and Sinigaglia, 2006). The first level involves simple motor schemas (e.g., finger flexions); the second level encompasses motor acts—sequences of movements aimed at specific goals (e.g., grasping food to eat); and the third level includes actions—sequences of motor acts aimed at general goals (e.g., eating).

To better understand specific systems, it is important to consider their gradients of complexity, automaticity (mandatoriness), sharing, and penetrability. As the number of assembled subsystems increases, a module becomes (i) more complex, (ii) less computationally encapsulated, and (iii) less automated. However, a module may still be considered as such if it reaches a certain level of expertise and mandatory operation.

At this point, the proposed model begins to take shape, emphasizing the gradient from automaticity to mandatory processing. This gradient is inversely proportional to modular complexity and is systematically organized according to the theory of Moscovitch and Umiltà’s (1990). This framework allows us to categorize learning processes that form modular systems, acting as a tool for distinguishing between first, second, and third types of modules.

The literature, however, suggests that the properties of modular systems extend beyond this framework. Further observations indicate that second and third-level modules, can transition from a modular to a non-modular state when controlled by executive attentional systems.

### 4.1 Interaction of neural networks in shifting between automatic and controlled states

The interplay between key neural networks is essential for shifting between automatic and controlled processes in the brain. Three main networks—the Default Mode Network (DMN), the Central Executive Network (CEN), and the Salience Network (SN)—coordinate this process.

**Figure 1:**
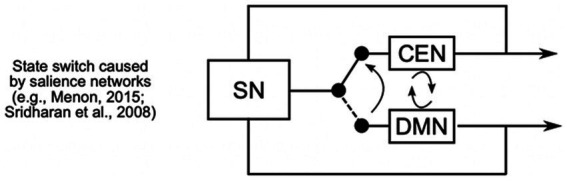
The Salience Networks (SN) play a crucial role in dynamically switching between the Central Executive Network (CEN) and the Default Mode Network (DMN). The SN recruits executive regions to maintain cognitive focus on task-relevant goals, while simultaneously suppressing DMN activity, which is typically associated with mind-wandering and self-referential thought (adapted from Menon, 2015).

The DMN, involving areas such as the precuneus/posterior cingulate cortex and medial prefrontal cortex, is responsible for internally focused thought, such as daydreaming or mind-wandering (Raichle et al., 2001). The CEN, which includes the dorsolateral prefrontal and posterior parietal cortices, is engaged when we need to focus on complex tasks requiring attention and problem-solving. The SN, primarily consisting of the anterior insula and anterior cingulate cortex, acts as a switch between these networks, evaluating the relevance of external and internal stimuli to determine whether the DMN or CEN should be activated (Dosenbach et al., 2007; Menon and Uddin, 2010; Sridharan et al., 2008). Figure 1 visually represents how the SN helps transition between automatic and controlled states by switching the brain’s focus from resting or mind-wandering (DMN) to active, goal-oriented tasks (CEN).

**Figure 2:**
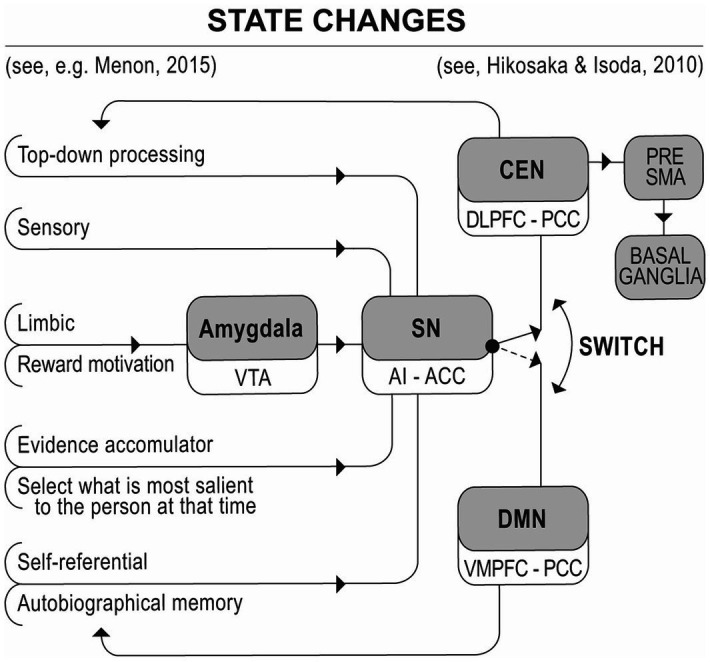
Sensory and limbic inputs processed, by the anterior insula, detect salient events, initiating control signals to regulate behavior and homeostatic states. This model, adapted from Menon (2015) and Hikosaka and Isoda (2010), illustrates the pathways through which salient stimuli are transformed into motor outputs via the Salience Network (SN) and Central Executive Network (CEN). It highlights how the SN, by processing significant environmental inputs, coordinates the activation of executive control systems to guide appropriate behavioral responses. VTA, Ventral Tegmental Area; AI, Anterior Insula; ACC, Anterior Cingulate Cortex; DLPFC, Dorsolateral Prefrontal Cortex; PCC, Posterior Cingulate Cortex; VMPFC, Ventromedial Prefrontal Cortex; PRE SMA, Pre-Supplementary Motor Area.

The SN functions as an “evidence accumulator” (Benso, 2018), triggering adaptive behavior once a certain threshold of a highly activated signal is reached. This mechanism engages or disengages the CEN based on the salience of incoming stimuli, filtering the most relevant signals for the context (Menon and Uddin, 2010; Petersen and Posner, 2012). The SN also connects with the amygdala and limbic centers, influencing behavior in typical situations (see Figure 2). For example, while walking is generally an automated process, in dangerous situations (e.g., hiking on a precarious trail), attentional executive systems closely monitor the activity, demonstrating a shift from DMN to CEN activation driven by emotional signals captured by the SN from the limbic centers.

Additionally, Hikosaka and Isoda (2010) described how circuits in the anterior insula, cingulate gyrus, pre-supplementary motor area, and basal ganglia facilitate switches from automatic to controlled actions, influenced by feedback or anticipatory signals. This shift is crucial for understanding how modular or non-modular systems are regulated by central systems and working memory. Figure 2 visually represents how sensory and limbic inputs contribute to regulating behavior and homeostasis. These interactions between the SN, CEN, and DMN highlight the complex interplay between cognition, emotion, and motivation. For instance, during a risky activity (e.g., climbing a via ferrata), the “walking” module may lose its automaticity due to anxiety detected by the SN, prompting the CEN to take control. Conversely, in a familiar, low-anxiety environment, an expert musician may perform a well-learned piece with ease, requiring minimal active CEN resources, as the music itself provides a salient focus.

In cases of emotional disturbances, such as depression or anxiety, these network interactions may become disrupted, leading to disengagement of the CEN when it is needed, or over-engagement when it is not (Benso, 2018).

**Figure 3:**
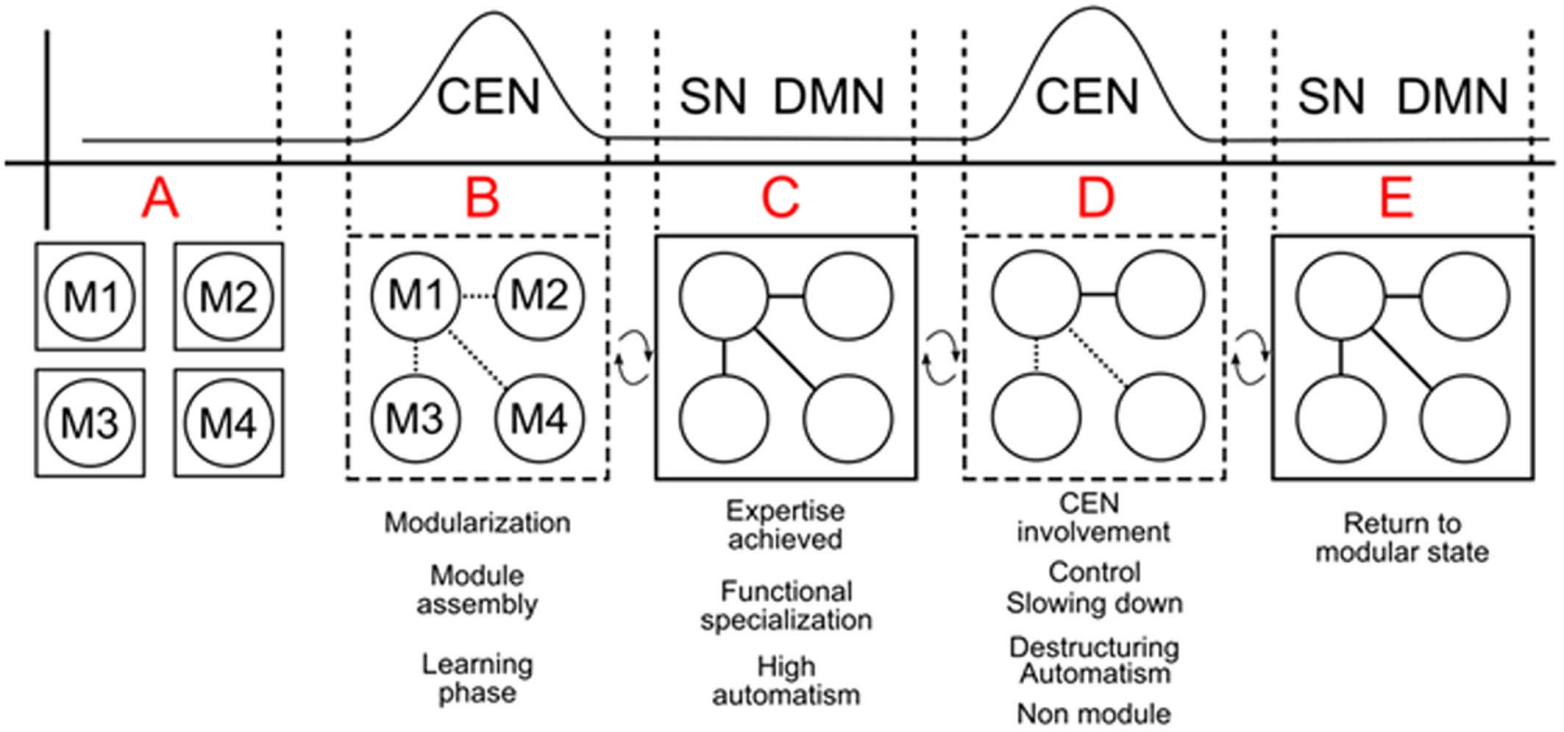
Outline of the Central Executive Network (CEN) in the creation and maintenance of complex systems through the assembly of various submodules (M1, M2, M3, M4) in phases A and B. Once a skill becomes modularized (phase C), the CEN’s control diminishes, and the Default Mode Network (DMN) or Salience Network (SN) assumes a lighter, more passive control. However, the CEN can re-engage (phase D) to modify or improve performance, such as during emotional regulation, performance training, or reconfiguring the system (e.g., when a pianist needs to adjust an established fingering pattern or when an athlete must fine-tune a motor skill). The system returns to a stable, routine modular state in phase E, where the modules operate efficiently without further engagement from the CEN. Dashed lines indicate phases where the submodules are not yet fully assembled into a complex system (phase A and B) or when a modularized system is partially deconstructed for control adjustments in phase D.

This disruption can manifest as performance issues, such as rigidity or disorganization, particularly in high-pressure situations like performing in front of an audience. Eckert et al. (2009) emphasize that heightened activity in the anterior insula and frontal opercular regions can impair the DLPFC’s ability to select optimal responses in individuals with elevated anxiety. Evaluating the processes of modularization and the achievement of expertise in complex systems, as illustrated in Figure 3, reveals that the role of working memory (WM) and the Central Executive Network (CEN) during the acquisition and automation of skills is well-documented (Buccino et al., 2004; Kelly and Garavan, 2005; Vogt et al., 2007). WM is heavily involved and must have sufficient resources to complete the phases of learning and modularization—phases A and B. In phase C, expertise is achieved, and the individual performs the hyper-learned skill directly and mandatorily. At this stage, the CEN is deactivated, allowing minimal control by the Salience Network (SN) or Default Mode Network (DMN). However, CEN control over the module can resume (phase D) in specific situations, as discussed earlier, whether due to emotional triggers (such as fear of falling or losing) or logically programmed decisions, as seen in the domains of dance, sports, and instrumental performance (see Vogt et al., 2007). In these fields, movements are deconstructed, slowed down, and new elements are repeatedly introduced to improve and enrich performance. Working memory remains crucial, requiring adequate resources to successfully navigate these phases of learning and modularization. In phase E, new expertise is attained, or the previous routine is re-established, and the mandatoriness of the process returns, allowing it to be considered a complex module once again.

Cowan et al. (2005) cite several studies demonstrating that weak WM is associated with difficulties in academic performance. Similarly, Pasqualotto et al. (2024) note that weak WM is correlated with difficulties in becoming proficient in reading. As expertise develops, CEN activity decreases, allowing for smoother, more automated performance (Jansma et al., 2001; Kelly and Garavan, 2005). However, in situations where the process is deconstructed, such as during initial learning, the lack of mandatoriness suggests that the system cannot be considered modular. During the re-engagement phase, new expertise or the return to a routine restores the mandatoriness of the process, which can now be considered a complex module again.

However, strong emotions or novel challenges can re-engage the CEN, disrupting the automated process and temporarily reducing modularity. Andrews-Hanna (2012) suggested that during the performance of cognitively demanding tasks, the CEN typically shows increased activation, whereas the Default Mode Network (DMN) shows decreased activation; the two networks are anti-correlated (Dosenbach et al., 2007; Menon and Uddin, 2010). Figure 3 illustrates a natural physiological process, showing how the CEN engages in the creation and adjustment of complex systems, highlighting the phases of modularization and re-engagement during novel or emotionally charged situations.

For a comparison of modular and non-modular states and their behavioral effects, refer to Table 4.

### 4.2 The limits of massive modularity in relation to central systems

There are numerous theoretical proposals for the modularization of central systems (see Barrett and Kurzban, 2006, for a review). However, despite the formation of certain circuits through hyperlearning in memory systems, it is crucial to differentiate between executive, attentional functions that are amodal and overarching from the dynamics of modular systems. Proponents of massive modularity, often rooted in the ideas of evolutionary psychology, tend to apply modularization even to central systems. This perspective has been met with significant criticism. For instance, Pietraszewski and Wertz (2022) argue that massive modularity itself is based on a misunderstanding regarding the appropriate level of analysis. Consequently, they suggest that the modularization of central systems is problematic. Similar to our work, these authors propose a stratified approach to avoid the misunderstandings that arise from conflating different degrees of modularity.

When developing a model for complex modules, it is important to consider the limitations of applying modularity to central systems. Central systems, while essential for the formation of complex modules, are not easily modularized or partitioned. These systems, as Lundie (2019) points out, likely evolved to remain flexible and adaptive rather than rigidly modular. He argues that central cognition, like belief formation and reasoning, is managed by coordinated domain-specific modules, with central systems adapting to brain physiology rather than being modularized. Further evidence against the modularization of central systems is provided by Cohen and D’Esposito (2016), who found a negative correlation between static modular organization and central cognitive task activation. This finding suggests that central systems may require a more integrative and dynamic architecture to support complex cognitive functions, rather than adhering to the rigid structures proposed by massive modularity. Recent studies by Yeon et al. (2024) utilizing fMRI have further contributed to this debate. They showed that the fronto-parietal network, supported by the CEN, exhibits general-domain functionality during the learning phases of various complex tasks. Barrett and Kurzban (2006) also argue that making central systems modular would lead to computational inefficiencies and potential failures in adaptive behavior. They suggest that if central systems were fully modularized, the resulting computational explosion could overwhelm cognitive resources, leading to suboptimal performance in complex tasks.

Despite these challenges, certain central faculties, such as aspects of semantic and procedural memory, do appear to modularize over time, becoming more automatic and less reliant on executive control (Moscovitch and Umiltà, 1990). For example, tasks such as braking while driving or answering routine questions (“What is the capital of France?”) can become highly automated. Various studies have explained the formation (though not the storage) of these memory circuits. Jonides et al. (2005), in their study on working memory, hypothesized that storage is mediated by the same brain structures that process perceptual information and that rehearsal engages a network of brain areas also involved in attention control.

Moscovitch (2008) provides another example of modular activity at the central level, focusing on memory. He describes how memory circuits, formed and consolidated by hippocampal activity, demonstrate a high degree of mandatoriness. Moscovitch’s proposal that the hippocampus functions as a “stupid” module dedicated to processing consciously apprehended information underscores the complexity of memory organization. This complexity involves a modular approach to certain memory functions, which may include episodic, semantic, and spatial memory, as well as the roles these memories play in perception, comprehension, planning, imagination, and problem-solving.

However, the executive attentional aspect remains at a higher, multifactorial, interactive, and amodal stage. Memory models incorporate executive attention and attentional control as necessary support during the phases of encoding, consolidation, and learning complex activities. Once expertise is reached, the executive attentional component withdraws, re-engaging only in specific situations, such as those involving emotional pressure (as suggested by the interaction between the Salience Network, Central Executive Network, and Default Mode Network; see also Figure 3).

The attempt to modularize executive attentional functions has often been criticized for being methodologically inappropriate and inconsistent (Rabbitt, 1997; MacLeod et al., 2003; Engle and Kane, 2004; McCabe et al., 2010; Hofmann et al., 2011; Benso, 2018). This criticism aligns with Rabbitt’s (1997, p. 10) statement on cognitive functions:

*“[…] it has passed unrecognized that hypothetical components of ‘executive behavior’ such as ‘inhibition’, ‘planning’, ‘monitoring’ and ‘control’, which are, in fact, simply descriptions of task demand, may have very poor construct validity because although these demands appear logically different they can be met by identical production system architectures”* (Rabbitt, 1997; p.10).

Despite the numerous publications that continue to perpetuate these differences, the reductionist approach in high-functioning systems remains questionable. The evidence suggests that while some central functions can achieve a degree of modularity, the inherent complexity and need for flexibility in central systems argue against a fully modular approach.

## 5 Dynamic modular systems: neuropsychological and behavioral perspectives

This paper proposes a reorganization of modular theory that integrates insights from multiple disciplines within the neurosciences. In this section, we introduce a comprehensive systematization that synthesizes interdisciplinary perspectives within the neurosciences, focusing on the dynamic interaction between modular and non-modular systems. This systematization is grounded in extensive behavioral and neuroimaging studies, reflecting the evolution of our understanding of modularity in complex cognitive systems over time.

This dynamic perspective extends beyond traditional Fodorian frameworks by incorporating modern concepts of functional specialization and cognitive flexibility. As a result, the three primary criteria traditionally used to confirm modularity have been redefined. In complex systems, “functional specialization” (Dehaene and Cohen, 2011) replaces the Fodorian notion of “domain specificity,” while encapsulation and mandatory operations (automaticity) are no longer interpreted as binary constructs. Instead, they exist on a continuum, with variation depending on system complexity and the level of expertise acquired through learning (Cohen et al., 1990; Petrov et al., 2010).

Crucially, the model presented here is not the result of discursive or opinion-based choices. Unlike many other modular theories, it is rigorously grounded in empirical research demonstrating robust experimental results. Although modularity theory is often subject to abstract theorization, we have prioritized empirical approaches, which inevitably stem from decisions shaped by the abductive reasoning inherent in experimental science. The data underpinning this systematization are derived from both our own experiments and an extensive body of literature.

The reorganization of modular theory responds to the critical needs identified in the literature. It offers an objective distinction between different degrees of modularity—innate, genetically predisposed, and acquired through hyper-learning supported by working memory. Furthermore, it explains complex modularity as a dynamic state rather than a fixed system, addressing the boundary between modular and non-modular systems. A system is considered modular when it operates with a certain degree of automaticity and mandatoriness. However, when regulated by the Central Executive Network (CEN), that same system may function as non-modular during more deliberate, slower processing. This theoretical framework also offers insights into well-established phenomena, such as separable modifiability (Sternberg, 2011) and double dissociations (Shallice, 1988), not only across subsystems but also among more complex systems, such as declarative and procedural memory (Purves et al., 2012). A key contribution of this framework is the recognition of state-dependent factors—driven by network activation—that determine the degree of modularity. This dynamic perspective accounts for both the stability and flexibility of cognitive systems, enabling them to adapt to different contextual demands.

Our approach has been informed by a thorough review of the literature, as well as experiences in clinical settings and insights from fields such as art and sports. Throughout this process, we employed abductive reasoning to select theories based on the strength of evidence, a method well-documented in scientific practice (Coltheart, 2011). Although it remains possible to create functional systems with mandatory characteristics within memory, memory itself must be understood in its broader, instrumental sense. As demonstrated in the example of hippocampal circuits described by Moscovitch (2008), memory systems can exhibit modular behavior. However, in line with modularization processes, central systems like the CEN play a supportive role during learning phases and gradually withdraw as expertise develops, re-engaging only when required by external demands or internal motivations (see Figure 3). This model emphasizes the modularity of memory systems formed through hyper-learning while maintaining the amodal nature of the CEN, which provides resources and support as necessary.

In summary, our systematization of modularity is the result of carefully considered decisions, guided by ongoing debates and a wealth of literature. These decisions were made to support and refine the concept of modularity within cognitive systems.

This organizational framework for modular theories, as inferred from the work presented thus far, involves a systematization of functional systems. These systems should be clearly distinguished through a taxonomic organization that avoids theoretical confusion, particularly when the term ‘module’ is used to compare realities of differing levels and complexities. By examining the schematic representations in Tables 3, 4, we can effectively organize these systems based on their complexity and various indices.

## 6 Implications

This systematization has significant implications for clinical practice, sports training, and educational settings. The modular models discussed within this framework are grounded in a broad body of literature that supports their practical relevance. We have extended these models to develop new experimental protocols and treatment approaches.

In terms of rehabilitation, embracing complex and dynamic modular models, as opposed to rigid, encapsulated systems, is more productive for practitioners. Such an approach allows for the breakdown of subsystems, stimulating multiple components of larger systems that may exhibit weaknesses. The concept of rigid, isolated systems, discourages rehabilitation efforts that should aim to penetrate and reintegrate subsystems. This perspective aligns with research showing that overly stereotypical and automated treatments can have detrimental effects on cognitive systems, particularly in terms of brain plasticity (Metzler-Baddeley et al., 2017). Their study, using Diffusion MRI techniques, demonstrated that non-adaptive, stereotyped treatments resulted in both cognitive regression and decreased white matter integrity in the CEN. In contrast, adaptive interventions, tailored to individual abilities, led to cognitive improvements and growth of new neural fibers in the same areas.

### 6.1 Integrated treatment model

The relationship between the model representing complex modularity (see Tables 3, 4) and the cognitive stimulation protocol outlined in Table 5, defined as Integrated Cognitive Training (ICT; Benso, 2004; Benso et al., 2021; Benso and Benso, 2023), is clearly established. Treatments aimed at improving complex modular activities that are underdeveloped or impaired due to trauma align closely with the modularity models presented here. The ICT incorporates the theoretical framework described above, which outlines the formation of complex modularity. It evaluates the stratification of levels, the potential subcomponents of the complex module, and the critical role of working memory and executive attention within the cognitive system being addressed. In essence, the proposed treatment model provides a comprehensive and integrated approach that targets both the modular components and the attentional and working memory systems involved in the development of a complex module. This approach is exemplified in the context of Specific Learning Disorders (see also Benso et al., 2021).

**Table 5:** Key steps in the integrated cognitive rehabilitation method (Benso et al., 2021).

Step	Description	Citation
1. Activation	Cognitive and motor “ready-set-go” activation exercises to engage attentional systems.	Robertson et al. (1998); Benso et al. (2021)
2. Specific Techniques	Tailored techniques based on the specific discipline (e.g., clinical, educational, sports)	Benso and Benso (2023)
3. Dual-Task Learning	Use of dual-task exercises to consolidate learning, deconstruct automatisms, and promote transfer.	Rypma and D’Esposito (2003); Mastropasqua et al. (2015)
4. Reinforcement of Working Memory	Intensive WM training through N-back, shifting, visual imagery, and dual-tasking, calibrated to the individual’s abilities.	Rypma and D’Esposito (2003); Benso and Benso (2023)
5. Personalized Difficulty Calibration	Tasks are precisely adjusted to the individual’s cognitive load and abilities, aiming to stimulate executive attention and maintain optimal learning conditions.	Benso and Benso (2023); Pasqualotto et al. (2023)
6. Role of the Human Operator	The presence of an empathetic, well-prepared operator is critical for improving treatment outcomes by maintaining motivation and attentional focus.	Diamond and Ling (2019); Sarter et al. (2006); Benso and Benso (2023)

The term integrated treatment refers to an approach that addresses both the specific module requiring recovery and the central systems (CEN) that need to be strengthened (Benso et al., 2021).

The ICT rehabilitation activities should begin with attentional *activation exercises*, such as alert tasks, designed to switch from the Default Mode Network (DMN) to the CEN, ensuring individuals are fully engaged before starting tasks. This shift also stimulates the Salience Network (SN), which plays a key role in regulating focus and engagement (Menon, 2015).

An important aspect of our model is the presence of an empathetic, human operator to foster motivation and emotional connection, as cognitive performance is often intertwined with emotional states (Diamond and Ling, 2019; Eckert et al., 2009; see section 4.1 and Table 4 for details on emotional-cognitive interactions). Motivation is a critical driver for sustaining attention (Sarter et al., 2006), and our treatment model prioritizes activities that target working memory through complex motor tasks, dual-tasking, visual imagery, task-switching (shifting), reworking in working memory, and n-back tasks. These exercises are highly effective in activating and strengthening CEN circuits, which are essential for the regulation and modulation of both cognitive and motor learning. Furthermore, dual-task exercises, when well-calibrated to the individual’s cognitive abilities, have been shown to accelerate learning, improve modularization, and enhance both the maintenance and transfer of skills (Rypma and D’Esposito, 2003; Mastropasqua et al., 2015; Varela-Vásquez et al., 2020). These exercises prevent over-reliance on automated responses and promote sustained engagement with the CEN, leading to more effective treatment outcomes. Our proposed integrated treatment model is built around key principles that guide the rehabilitation process. These principles, outlined in Table 5, provide a detailed overview of the core components and associated protocols. For further details, refer to Benso et al. (2021) and Benso and Benso (2023). For instance, Ciarmiello et al. (2015), applying this treatment, demonstrated significant cognitive and neural improvements (as evidenced by PET imaging) in amnesic MCI subjects, the profiles of the treatments used have been better explained in a subsequent publication by Benso et al. (2021). Similarly, Veneroso et al. (2018) applied modular principles to educational interventions, incorporating activation exercises during lessons and intervals with games and tasks aimed at attentional engagement and reinforcement of working memory, showing significant gains in reading, writing, and attention among children in the experimental group. These findings underscore the importance of integrating modular components and attentional systems in rehabilitation strategies.

### 6.2 Specific learning disorder: incomplete modularization

The modular framework provides a valuable lens for understanding different disorders, including Specific Learning Disorders (SLD), such as developmental dyslexia and dysgraphia, by conceptualizing them as incomplete modularization processes. For instance, Moscovitch and Umiltà’s (1990) modular framework for reading integrates second-type modules, combining linguistic and visuospatial systems supported by executive attention and working memory through hyperlearning. Predictive tests for reading difficulties, developed based on this model, assess key predictors such as visuoconstruction, phonological awareness, and working memory resources (Benso et al., 2019). This approach is aligned with national guidelines on the diagnosis of reading disorders.

In dyslexia, mandatoriness (the automaticity of reading) is significantly uncertain and underdeveloped, which forces the individual to rely on limited working memory resources, hindering comprehension (Pasqualotto et al., 2024). Training protocols that target the components of reading (e.g., phonological awareness, spatial attention, working memory, executive attention) improve reading outcomes by fostering the automaticity of these processes (Benso et al., 2021). This highlights the utility of a stratified modular model for addressing SLDs, where submodules such as linguistic and visuospatial systems are treated alongside working memory (see Tables 3, 4).

For dysgraphia, the interplay between working memory, visuospatial processing, and fine motor control creates challenges in automaticity. The individual must rely heavily on working memory to control handwriting, which leaves insufficient resources for accuracy checking. Teachers need to understand that this incomplete modularization can lead to frequent spelling errors when too much attention is devoted to motor control. Our proposed training model integrates attentional interventions that target both the visuomotor and cognitive systems to improve handwriting (Benso et al., 2021).

Furthermore, various disorders known in neuropsychology are explained and organized by the Moscovitch and Umiltà (1990) model for each modular level (see a concise exemplification of neuropsychological disorders in Table 3).

### 6.3 Broader clinical applications

Recent advances in neurocognitive research have highlighted the critical role of salience network (SN) disruptions in disorders such as autism, schizophrenia, and frontotemporal dementia. These disruptions interfere with the coordination between the SN and the Central Executive Network (CEN), impacting cognitive and emotional processing. For instance, Uddin (2015) demonstrated that neural signals originating from the insula could accurately assist in diagnosing autism, providing a neurobiological basis for understanding the disorder. Interventions that focus on improving SN-CEN coordination, such as real-time social decision-making tasks, have shown potential in reinforcing the salience of social cues, ultimately enhancing social functioning in individuals with autism (Uddin, 2015, 2021; Pasqualotto et al., 2021). Further research into SN-CEN interactions could inform targeted therapeutic strategies for other disorders characterized by similar network disruptions, such as schizophrenia and frontotemporal dementia. For additional details on how SN-CEN coordination influences cognitive and emotional processes, see Table 4.

## 7 Future directions

The distinction between modular and non-modular systems, and their implications for functional specialization, remains a critical area for further exploration. A key question is whether cognitive tasks that heavily depend on executive control—such as phonological awareness tests (e.g., the spoonerism test)—accurately represent the cognitive domain they are designed to measure. While these tasks are traditionally seen as measures of linguistic ability, they also engage broader cognitive processes, including working memory and executive attention (Franceschini et al., 2012; Varvara et al., 2014; Knoop-van Campen et al., 2018; Pasqualotto et al., 2024).

Given that tasks like the spoonerism test rely heavily on executive control, the boundaries between modular systems and non-modular executive processes need further investigation. Exploring how executive functions might overshadow or interact with modular cognitive functions can provide insights into refining diagnostic tools, particularly for conditions like dyslexia.

Additionally, we suggest that future research should focus on developing new measurement tools that evaluate the integrity of specific cognitive networks. These tools could help distinguish deficits in executive attention and working memory that stem from different interacting systems, enabling more precise patient profiles and better-targeted interventions. While some tools for assessing the CEN exist (as mentioned in the broader article), there is a lack of tools to evaluate the efficiency of the SN, which plays a crucial role in attention (Menon, 2015).

Finally, a shift toward technology-based assessments offers a promising avenue for improving detection and intervention strategies. With real-time measurement of cognitive functions and executive processes, these tools could enhance diagnostic precision, facilitate early intervention, and allow for more personalized treatment plans (Henkel et al., 2024). This shift could have profound implications for clinical practice, making it easier to tailor interventions based on individual cognitive profiles. An additional diagnostic challenge arises from the fact that potential CEN weaknesses may not necessarily involve attentional systems but could instead result from depressive or obsessive tendencies or intrusive thoughts that disrupt a subject’s performance during the DMN state. Alternatively, as a third hypothesis, the SN might fail to adequately activate the CEN during the test. These three hypotheses, which emerge in cases of poor performance on tests evaluating CEN (executive attention and working memory), can only be addressed by expanding anamnesis sessions and collecting much broader information about the subject (Benso et al., 2021). In the near future, these challenges could potentially be addressed by leveraging AI-driven diagnostic systems. For instance, advanced AI programs could integrate multimodal data—such as behavioral, physiological, and test performance metrics—to enhance diagnostic accuracy and propose personalized interventions.

In conclusion, this systematization of modular models not only integrates existing neuropsychological and behavioral research but also offers a comprehensive framework for understanding modularity in complex cognitive systems. By applying this dynamic modular framework to clinical practice, more precise diagnoses and better-tailored interventions can be developed, ensuring improved outcomes for individuals with a wide range of cognitive and neuropsychological disorders (Karbach et al., 2015; Loosli et al., 2012; Benso, 2018; Pasqualotto and Venuti, 2020; Benso et al., 2021; Pasqualotto et al., 2022; Benso and Chiorri, 2023).
